# Supplementary material for: Tumor-selective catalytic nanomedicine by nanocatalyst delivery
Source: Nat Commun. 2017 Aug 25;8:357. doi: 10.1038/s41467-017-00424-8 (PMC5572465; doi:10.1038/s41467-017-00424-8)
Supplement: Supplementary file 1 — Supplementary Information [file 41467_2017_424_MOESM1_ESM.pdf]

Description of Supplementary Files

File Name: Supplementary Information
Description: Supplementary Figures and Supplementary References

File Name: Peer Review File

Supplementary Figures

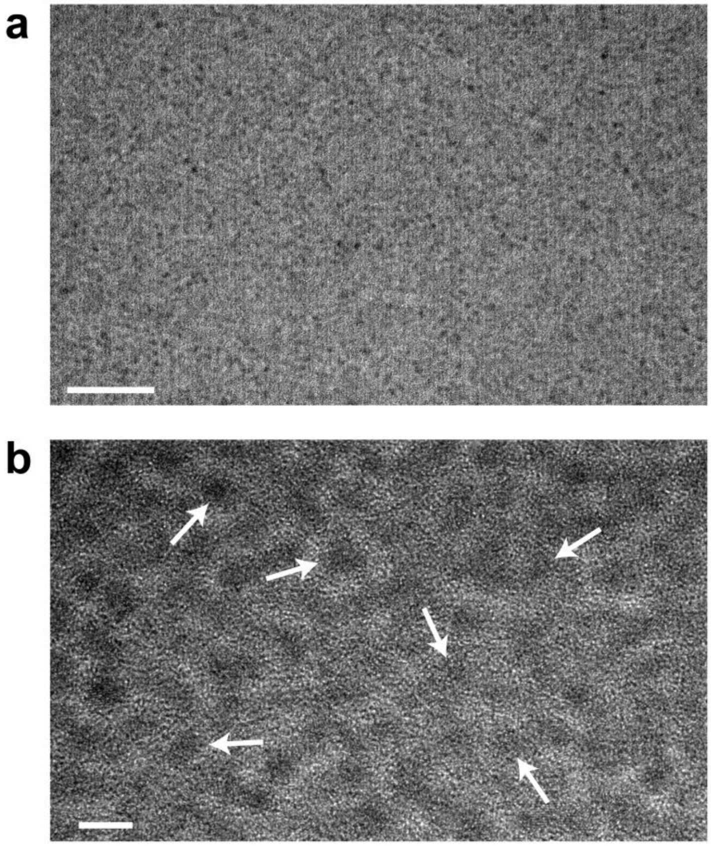

Supplementary Figure 1. a-b) TEM images of as-synthesized ultrasmall Fe_3O_4 nanoparticles. Arrows indicate the nanoparticles. Scale bar: a: 50 nm; b: 5 nm.

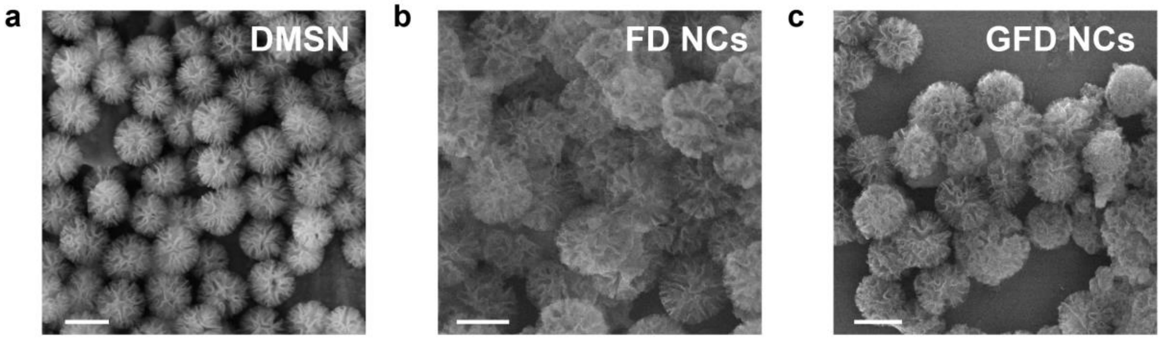

Supplementary Figure 2. SEM images of a) DMSN, b) FD NCs and c) GFD NCs. Scale bar: 200 nm.

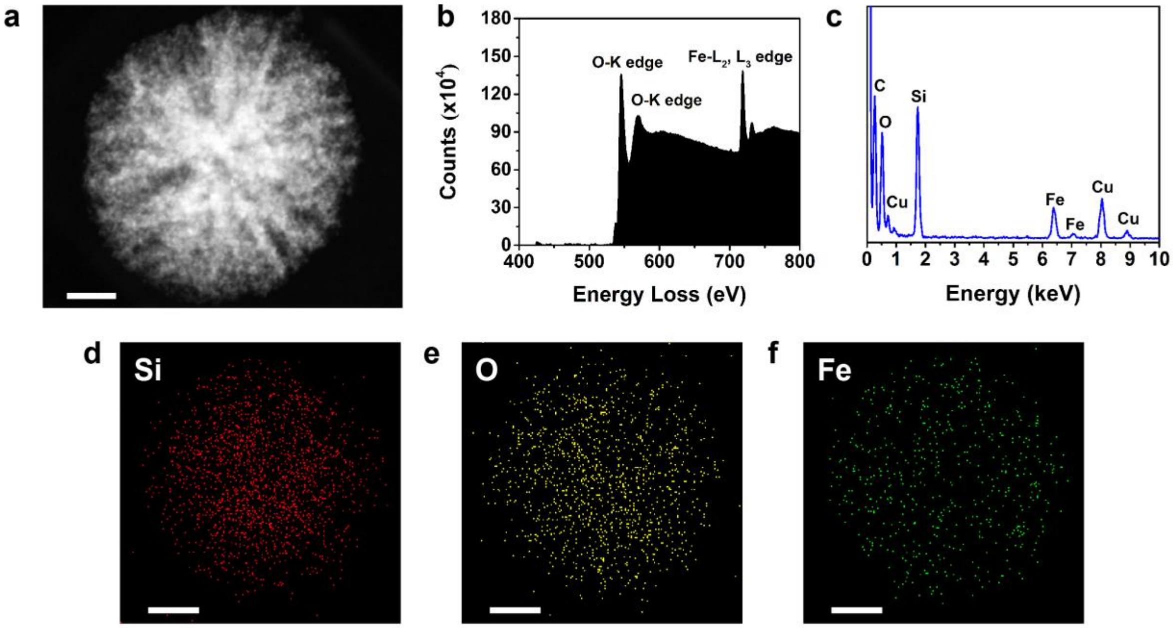

Supplementary Figure 3. a) Dark field image, b) electron energy loss spectrum, c) energy dispersive spectrum and d-f) corresponding elemental mapping of FD NCs. Scale bar: a, d, e, f: 50 nm.

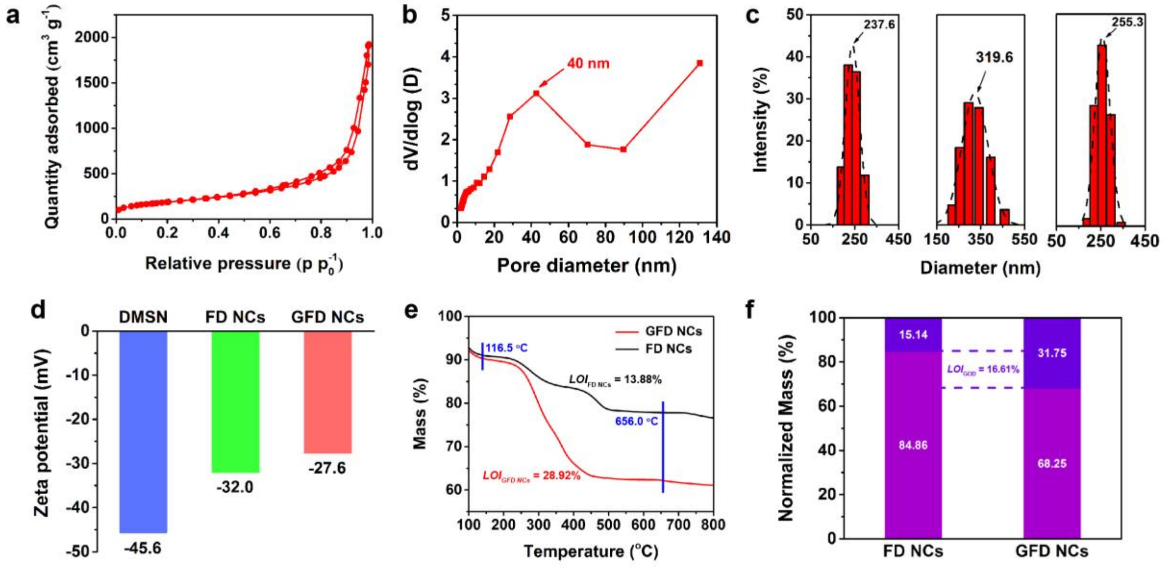

Supplementary Figure 4. a) N_2 adsorption-desorption isotherms and b) pore size distribution of DMSN supports. c) Hydrodynamic diameter (D_h) and d) Zeta potential of DMSN supports, FD NCs and GFD NCs respectively. e) Thermogravimetric (TG) curves and f) normalized weight loss distribution diagram of GFD NCs and FD NCs (Temperature range: 100 ^{o}C - 800 ^{o}C, ramping rate: 10 ^{o}C per min). The loss-on-ignition (LOI) of GFD NCs and FD NCs were 13.88 % and 28.92 % within the selected temperature interval from 116.5 ^{o}C to 656.0 ^{o}C. The LOI of FD NCs mainly originated from the ignition of organic functional groups on the surface of Fe_3O_4 NPs such as oleic acid. [1] Compared to the LOI of GFD NCs, additional GOD were burnt out. Thereby the loading amount of GOD could be calculated by the discrepancy of normalized LOIs between GFD NCs and FD NCs. The result turned out that the loading amount of GOD in GFD NCs was 16.61 %.

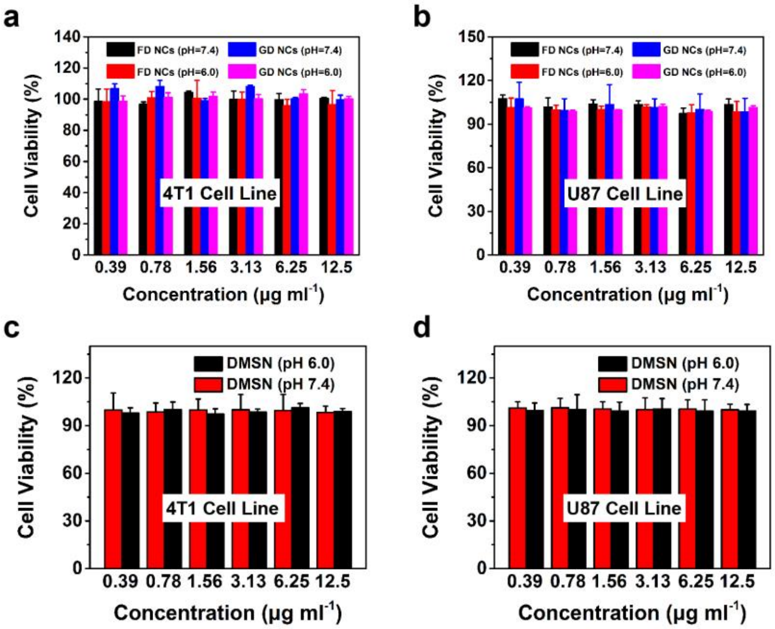

Supplementary Figure 5. *In vitro* cytotoxicity profiles of FD NCs, GD NCs and DMSN under neutral (pH = 7.4) and acidic (pH = 6.0) conditions. Mean values and error bars are defined as mean and s.d., respectively. Experiments were performed in quadruplicate.

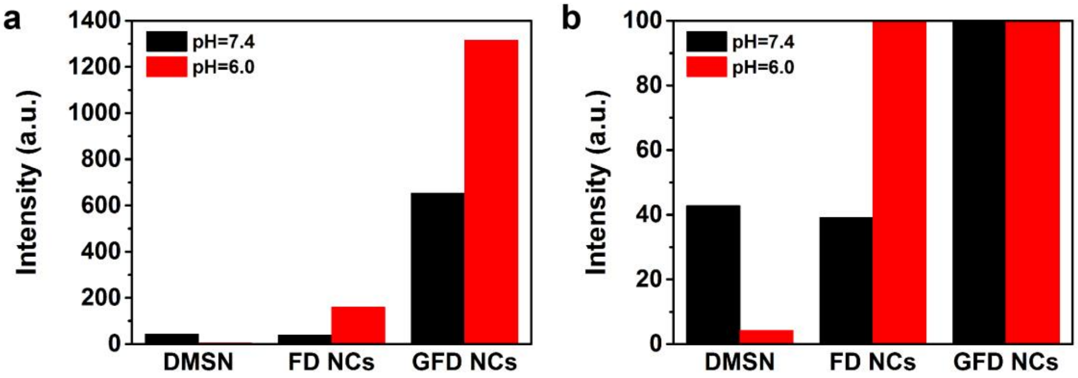

Supplementary Figure 6. a) Intensity profiles of confocal images in **Figure 4f** representing ROS generation. b) Enlarged scale image of a). Under acidic conditions (pH = 6.0), the green fluorescence intensity of GFD NCs was about 7 folds higher than that of FD NCs, and about 280 folds than that of DMSN supports. As for ROS-indication under neutral condition (pH = 7.4), the fluorescence intensity was overall dramatically lower. These profiles quantitatively present the efficacy of radical production under varied conditions.

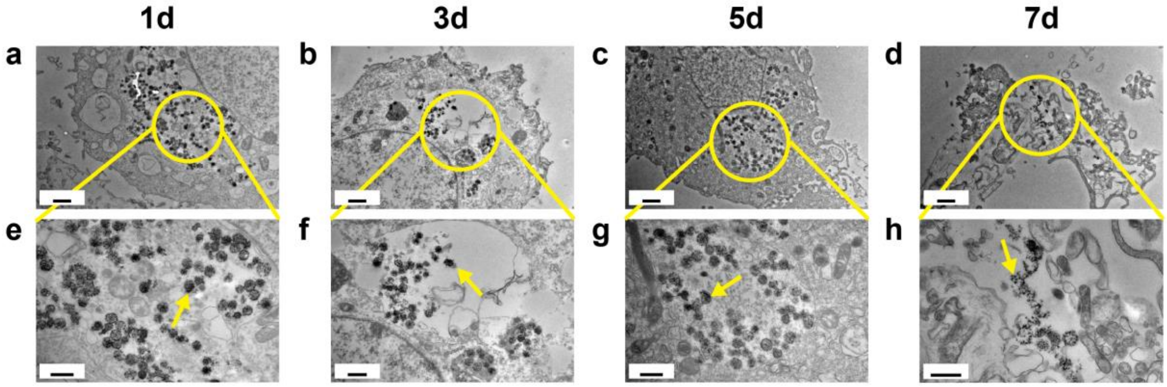

Supplementary Figure 7. Bio-TEM images demonstrating the intracellular degradation behavior and structure evolution of GFD NCs after co-incubation with 4T1 cells for a-d) 1 d; 3 d; 5 d and 7 d. Scale bar: 1 μm. e-h) Corresponding magnified images of the circled regions. Yellow circles indicate the endocytosized GFD NCs. Scale bar: 0.5 μm.

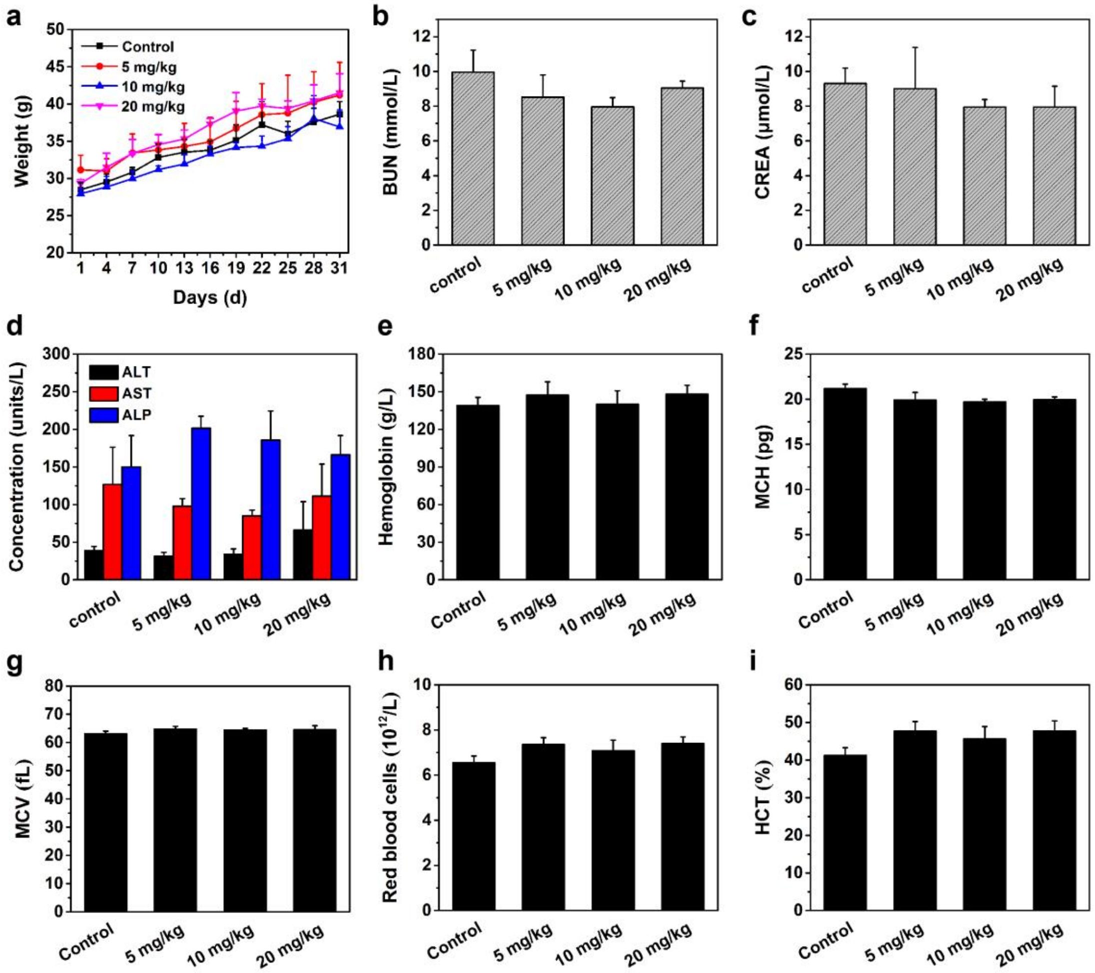

Supplementary Figure 8. a) Body weights of mice treated with saline (Control), 5 mg/kg, 10 mg/kg and 20 mg/kg of GFD NCs (Experiment), recorded every three days and within one month evaluation period. b) BUN level, c) CREA levels, d) ALT, AST and ALP levels of mice in all groups, reflecting the kidney functions and liver functions. e) Hemoglobin, f) MCH, g) MCV, RBC and i) HCT levels of mice in all groups. Mean values and error bars are defined as mean and s.d., respectively.

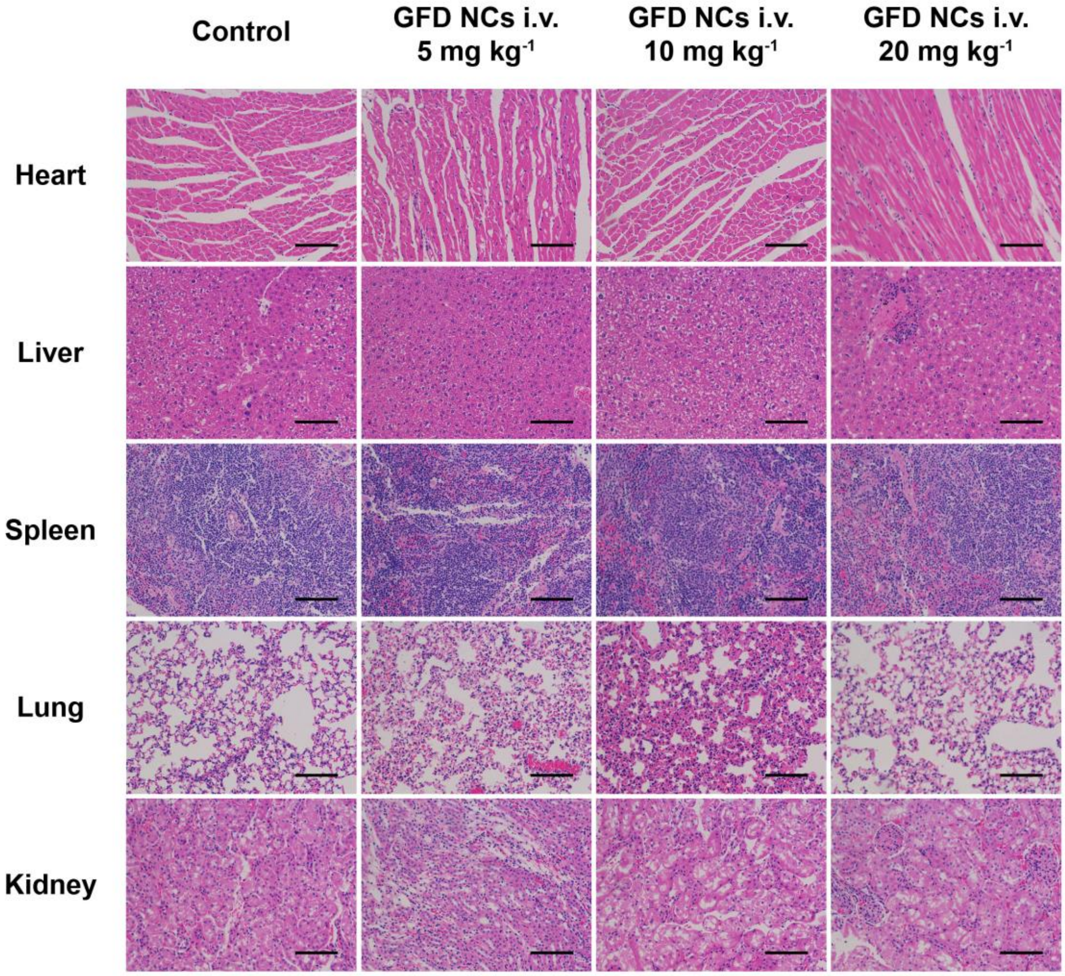

Supplementary Figure 9. Histopathology images of dissected major organs (heart, liver, spleen, lung and kidney) stained with H&E of control and all experiment groups for *in vivo* biosafety evaluation. Scale bar: 100 μm.

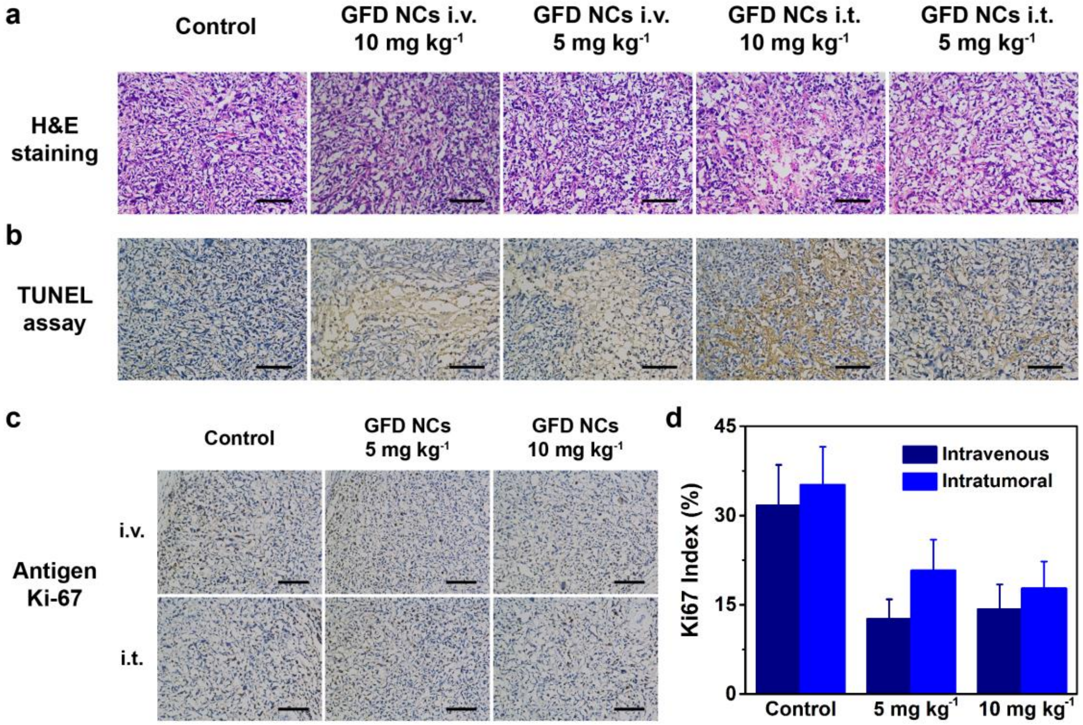

Supplementary Figure 10. Histopathology images of dissected 4T1 tumor xenografts stained with a) H&E, b) TUNEL assay and c) Antigen Ki-67. Scale bar: 100 μm. d) Ki-67 index of the corresponding images calculated in ImageJ software. Mean values and error bars are defined as mean and s.d., respectively.

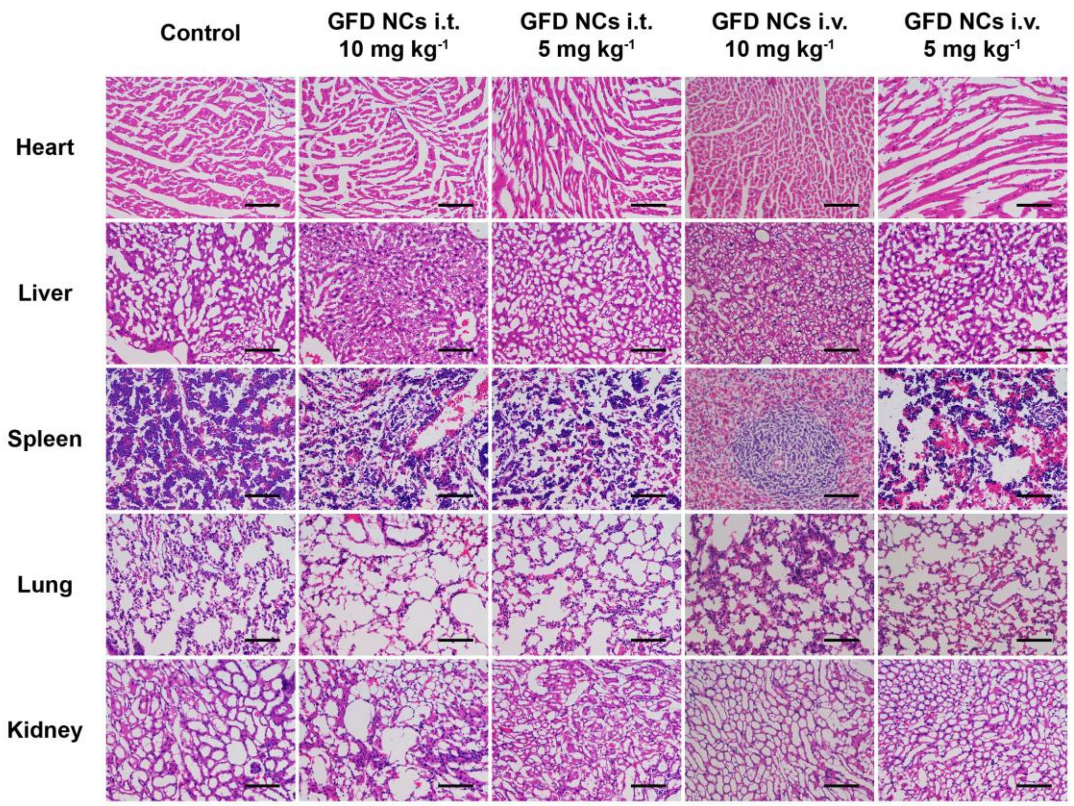

Supplementary Figure 11. Histopathology images of dissected major organs (heart, liver, spleen, lung and kidney) stained with H&E of control and all experiment groups for *in vivo* therapeutic performance experiment in 4T1 tumor xenograft. Scale bar: 100 μm.

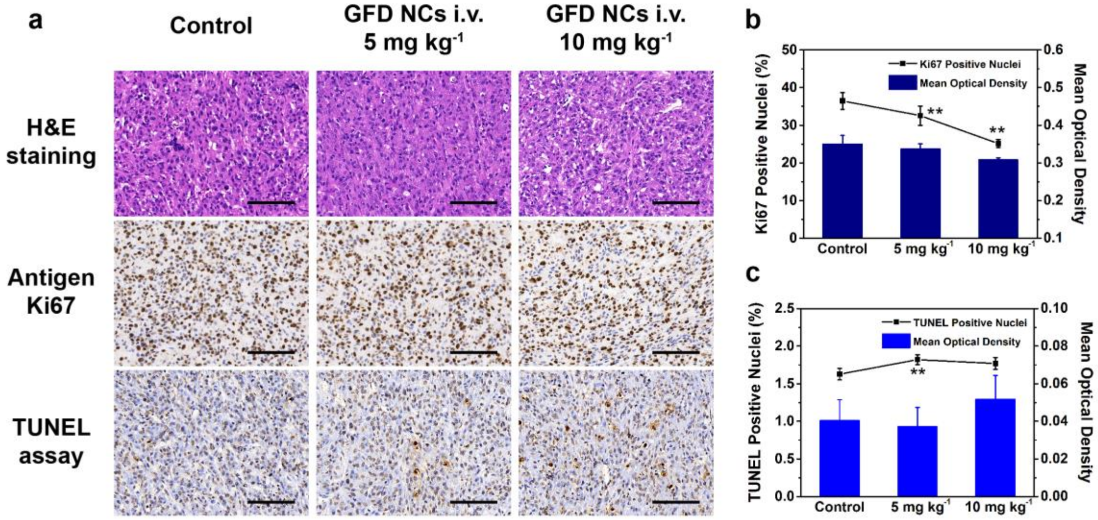

Supplementary Figure 12. Histopathology images of dissected U87 tumor xenografts stained with a) H&E, TUNEL assay and Antigen Ki-67. Scale bar: 100 μm. b) Ki-67 index and mean optical density of positive area of Ki-67 images. c) TUNEL positive nucleus and mean optical density of positive area of TUNEL images. Quantification of these histopathology images were calculated in ImageJ software. Statistical significance is assessed by Student's two-sided *t*-test compared to the control group. **$p<0.01$. Mean values and error bars are defined as mean and s.d., respectively.

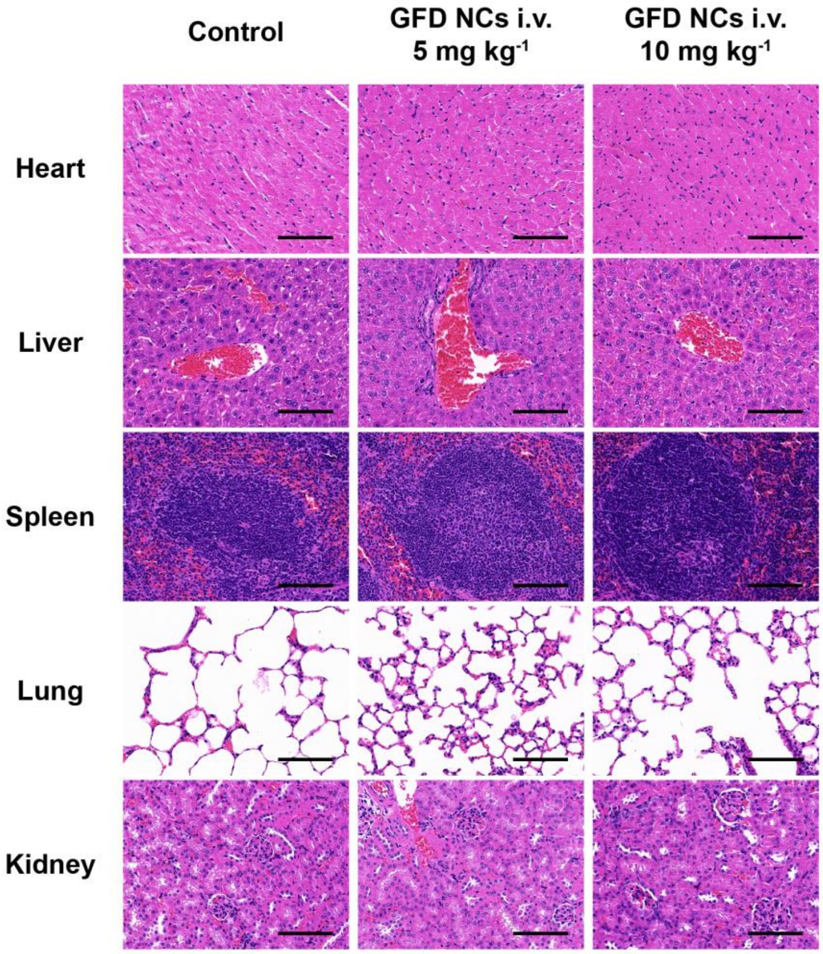

Supplementary Figure 13. Histopathology images of dissected major organs (heart, liver, spleen, lung and kidney) stained with H&E of control and all experiment groups for *in vivo* therapeutic performance experiment in U87 tumor xenograft. Scale bar: 100 μm.

Supplementary References

1. Sun S. *et al.* Monodisperse MFe_2O_4 (M = Fe, Co, Mn) nanoparticles. *J Am Chem Soc* **126,** 273-279 (2004).
